# Development and performance of a new prosthesis system using ultrasonic sensor for wrist movements: a preliminary study

**DOI:** 10.1186/1475-925X-13-49

**Published:** 2014-04-23

**Authors:** Nasrul Anuar Abd Razak, Noor Azuan Abu Osman, Hossein Gholizadeh, Sadeeq Ali

**Affiliations:** 1 Department of Biomedical Engineering, Faculty of Engineering, University of Malaya, Kuala Lumpur 50603, Malaysia

**Keywords:** Anthropometrics in designing prosthesis, Biomechatronics in prosthesis, Motion analysis, Transradial prosthetics

## Abstract

**Background:**

The design and performance of a new development prosthesis system known as biomechatronics wrist prosthesis is presented in this paper. The prosthesis system was implemented by replacing the Bowden tension cable of body powered prosthesis system using two ultrasonic sensors, two servo motors and microcontroller inside the prosthesis hand for transradial user.

**Methods:**

The system components and hand prototypes involve the anthropometry, CAD design and prototyping, biomechatronics engineering together with the prosthetics. The modeler construction of the system develop allows the ultrasonic sensors that are placed on the shoulder to generate the wrist movement of the prosthesis. The kinematics of wrist movement, which are the pronation/supination and flexion/extension were tested using the motion analysis and general motion of human hand were compared. The study also evaluated the require degree of detection for the input of the ultrasonic sensor to generate the wrist movements.

**Results:**

The values collected by the vicon motion analysis for biomechatronics prosthesis system were reliable to do the common tasks in daily life. The degree of the head needed to bend to give the full input wave was about 45° - 55° of rotation or about 14 cm – 16 cm. The biomechatronics wrist prosthesis gave higher degree of rotation to do the daily tasks but did not achieve the maximum degree of rotation.

**Conclusion:**

The new development of using sensor and actuator in generating the wrist movements will be interesting for used list in medicine, robotics technology, rehabilitations, prosthetics and orthotics.

## Background

Functional prosthetic hands can be classified into two parts; body powered prosthesis (uses tension cable) and externally powered prosthesis (electrically powered). Body powered prosthesis has a few other types based on need of the amputees [1-4]. The advantage of this type of prosthesis is the same like other types of prosthesis such as it is moderate in terms of its cost and weight. Other than that, it has high sensory feedback and easy to be learnt. The disadvantages of this type of prosthesis are it is not cosmetically very well and the user needs to teach some gross limb movements [5]. Usually the movements require high force at the shoulder to pull the tension cable (known as Bowden cable) until the movement of the task are assigned. The common tasks that can be done by this body powered prosthesis are usually pick and place which involved the movement of the wrist part namely pronation/supination and flexion/extension [6-11].

From the statistics values shown in many research, the transradial cases is the major population compared to other types of amputation cases [12-14]. Moreover, researchers also found that quite a number of amputees have reported the low wearing period of prosthesis with the dissatisfaction in terms of low functional in community and annual daily life activities (ADLs), cosmetic appearance and the discomfort of harness [2]. Supination/pronation and flexion/extension of the wrist are the frequently used motion in our daily life and thus the evaluation towards these motions is necessary to be carried out for better improvement. The combination of prosthesis motion with the compatibility of the subject by worn it is the important part need to be considered in this study.

Biomechatronics had made the combination of robotics and prosthetics become together. The system usually operated by applying input from sensor that will transmit the data to the controller to move the DC or servo motor [5,15-20]. Brain – computer interface (BCI) using the input from the brain to transmitted the input [21,22], while Pneumatic glove using the nerve from the amputation level to get data [19].

**Figure 1:**
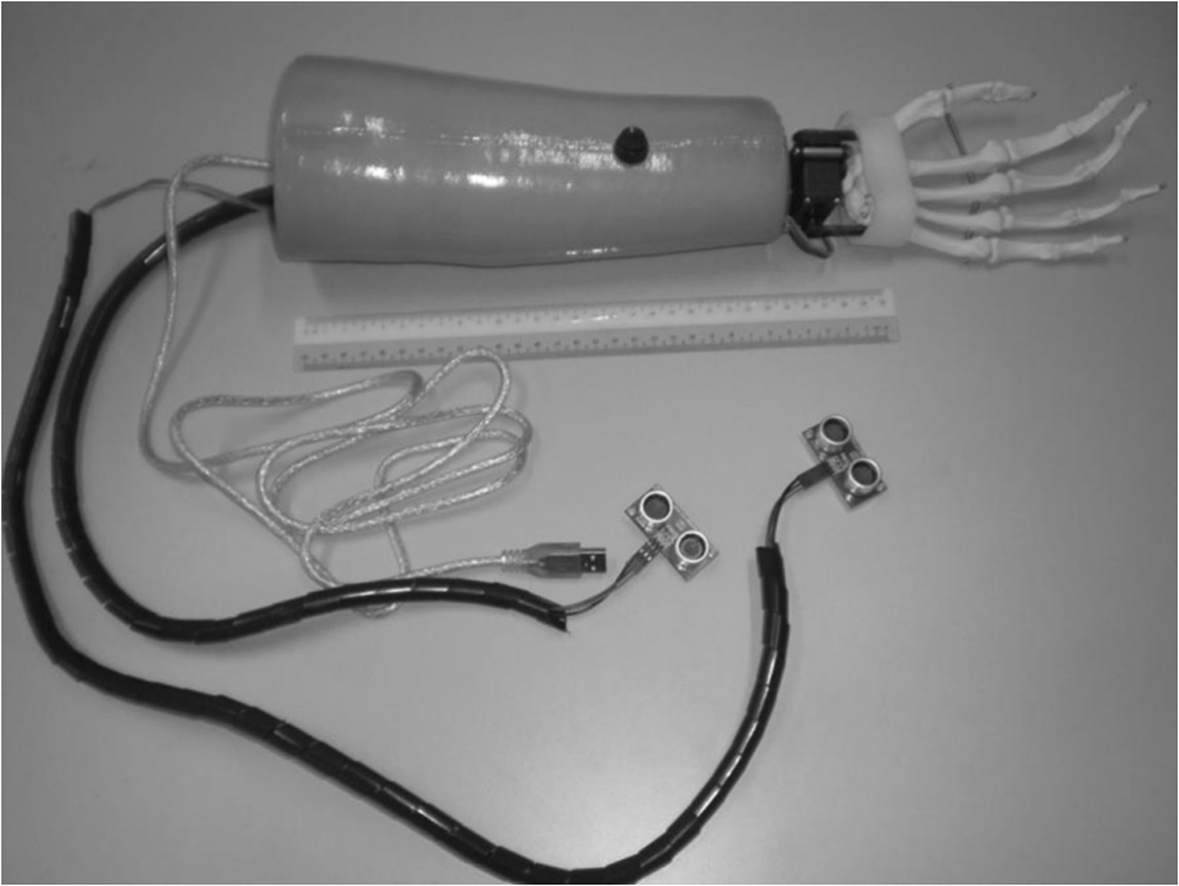
New development of the biomechatronics wrist prosthesi using ultrasonic sensors as the input, servo motors as the output and microcontroller.

Current robotics technology is moving synchronously with this prosthetics area. Both body powered prosthesis and also externally prosthesis can be combining together into one system in order to bring a new development in the prosthetics technology. This paper presents the new development of prosthesis system that used ultrasonic sensors as the replacement for the Bowden tension cable. The system was implemented by two ultrasonic sensors, two servo motors and microcontroller inside the prosthetics hand for transradial user (refer Figure 1). The paper will discuss the method of motion between the shoulder’s ultrasonic sensor and the head, the anthropometry of transradial prosthetic designs, and experimental performance of each wrist movements that using servo as an actuator. The related study has been described by the authors in previous studies [23,24].

## Methods

### Biomechatronics wrist prosthesis

**Figure 2:**
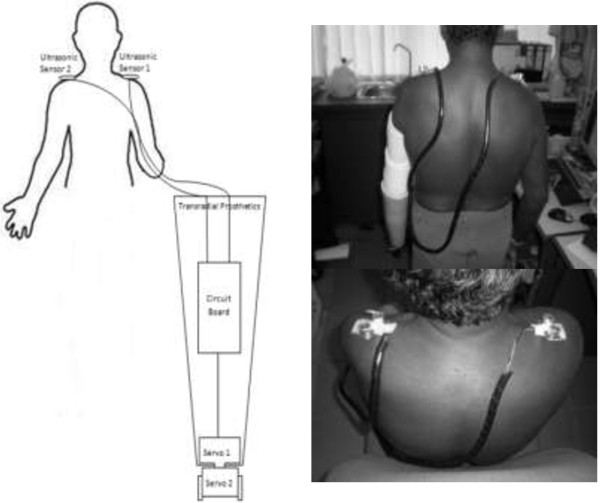
**Structure of the new biomechatronics wrist prosthesis.** Noted that the ultrasonic sensors were attached on the shoulder to rotate the servo motor placed inside the prosthesis hand.

The biomechatronics wrist prosthesis hand basically used ultrasonic sensor to transfer any motion detection data to the microprocessor and microcontroller-based system. The ultrasonic sensor is one of the most accurate and reliable measurement tools to determine human motion intensity [7]. An ultrasonic sensor used the transmitted and received wave to get the reflection of any motion within 0-15 cm. The sensor is attached to the amputee’s shoulder to replace the tension cable in body powered prosthesis [11,23,24]. The full figure of the mechanism is shown in Figure 2, instead of using only motion detection; the patient does not have to worry about training his muscle movement to operate the system as compared to the body powered that using the tension cable.

The Arduino microcontroller circuit functions as the main controller of the system that will control the main input and output of the system. The microcontroller is chosen for its ability to determine any type of output/input system either in analogue or digital form. Besides, it has 14 digital input/output pins (of which 6 can be used as PWM outputs), 6 analogue inputs, a 16 MHz crystal oscillator, a USB connection, a power jack, an ICSP header, and a reset button. The programme is stored in ROM (read only memory) and generally does not change. A microcontroller also takes input from the device and controls the device by sending signals to different components in the device. It contains everything needed to support the microcontroller; simply connect it to a computer with a USB cable or plug it with a AC-to-DC adapter or battery to get started. The advantage of using Arduino microcontroller is that the program memory is integral to the chip and it is small in size, which is also due to its on-board memory.

The sensor that functions as the input will then generate the data into the microcontroller system that is placed inside the transradial part. This part of the transradial also consists of two servo motors that operate as the replacement of motion of the extension/flexion and supination/pronation movements. The servo motor also has its degree of rotation limit similar to the wrist movement of the biological human hand. Servo motor is able to generate a maximum of 30 Nm of torque, which is greater than the required power to do daily tasks that usually need only around 10-30 Nm [1]. Servo 1 can generate the pronation/supination movement while Servo 2 is used in flexion/extension movement. The power supply for the system comes from the 9 V batteries that is well-known because it is very light in weight and long lasting. Servo motor is used as the output of the prosthetics system. There are a few types of motor used to design a robotic system. Some common examples are the servo motor, stepper motor and DC motor. All of them have their own classifications, advantages and disadvantages. But, the most common motor used in robotic system is usually the servo motor. Besides having high torque, servo motor has a capability to rotate precisely according to the degree assigned. For this system, the capability of the servo to generate the motion is required. The chosen maximum torque that can be applied by the servo is 13Nm. This is because in designing the prosthetics hand, it needs to deal with the high rate motion such as to pick and place motion and rotation motion.

### Protocol approval

The subject was selected from the University Malaya Medical Centre (UMMC), Kuala Lumpur. The inclusion criteria consist of a minimum 12 cm residual limb length (from the radii and ulna bone until end of residual limb), no wound and ulcers in the residual limb, and the ability to flexion/extension of elbow without the use of assistive devices. The subject was also considered for participation if they had used prosthesis in the last 2 years. All human test protocols were approved by the University of Malaya Medical Centre Ethics committee, and subject’s written informed consent was obtained before data collection.

### Anthropometry height analysis

The uses of anthropometry are to study the physical measurement of the human body by classifying them into few classifications such as sex, weight, height, and age. Most of these needs are satisfied by basic linear, area and volume measures [25]. However, human body motion usually requires more specific data such as the torque, force, angular velocity and man power.

Based on Drillis and Contini theorem [23,24,26], the body segment length can be defined according to the measurement of its height. Then the height will be multiply accordingly to each segment index for example 0.146 H for the transradial part [26]. In order to design the transradial prosthetics that is suitable for Malaysian, the full data of average Malaysians’ heights and weights were collected from the Kuala Lumpur Hospital (HKL) in 2000 [27]. The data show that the average of human height in Malaysia is 1.64 m for male and 1.53 m for female.

The data were measured among 200 adults in Malaysia with the age from 20 and above. Table 1 show that the transradial segment of a normal human is about 0.24 m for male and 0.22 m for female. These values are the transradial part that covers from the below elbow until the hand. But, to design the transradial prosthetic hand, the range should be lower than those values since the patient needs to slot in the remained residual limb into the socket. The length of the transradial prosthetic hand is very important when we deal about the balancing and stability of human body movement. The length must be suitable to be worn by the amputee and synchronous with the length of the other side of the hand. Otherwise, the hand would look awkward and is not comfortable to be worn.

**Table 1:** **Average height and segment length of hand for Malaysian population**[27]

	**Average male height (m)**	**Average female height (m)**	**Sample/population/age range**	**Methodology**
	**1.65**	**1.53**	**20+**	**measured**
**Length of segment**	**Shoulder (m)**	**Transhumeral (m)**	**Transradial (m)**	**Hand (m)**
Male	0.21	0.31	0.24	0.18
Female	0.20	0.29	0.22	0.17

### Anthropometry mass analysis

**Figure 3:**
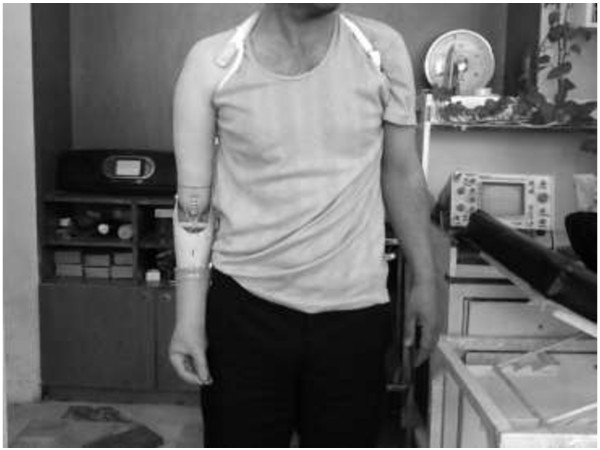
Sample of Imbalance body structure.

Human body segment has a lot of criteria to be considered especially the balance of the cross-section between the left and right sides of the body. The left side of the body usually has almost similar mass with the right side of the body [26]. This is the main criterion for human body mass because it involves walking, running, moving and even standing. If the body segment is imbalanced, it would interrupt the body movement and the other side of the body needs to be trained to cope with the imbalance. Figure 3 show example of imbalance body structure cause by wearing heavier prosthetic. The mass for the transradial segment can be calculated by multiplying the total mass of the human body with 0.00160. The usual mass of the transradial part for Malaysian male in average is about 0.95 kg [27].

Table 2 shows the mass that need to be considered to maintain the movement of the amputee and make it feels comfortable by wearing the transradial prosthetics. The details of the actuator mass are also shown in the Table 2. The outer part of the design uses the polypropylene materials and plaster of paris bandage that give the total mass of the design to be only about 0.69 kg. The value is much lesser compared to the exact mass of human hand that is about 0.95 kg.

**Table 2:** Mass distribution of the biomechatronics wrist prosthesis

	**Mass of 1 unit**	**No of unit**	**Total mass**	**Overall mass**
	**(kg)**		**(kg)**	**(kg)**
Biomechatronics wrist design				
Servo motor	0.06	2	0.12	
Arduino circuit board	0.03	1	0.03	
9V battery	0.03	1	0.03	
6V battery	0.01	1	0.01	
Wiring	0.01		0.01	
Interior prosthetics			0.19	
Exterior prosthetics	0.50		0.50	
Prosthetics				0.69
Normal male Hand				0.36
Normal male Hand + Forearm				1.31
Transradial Prosthetics + Hand				1.05

By using anthropometrics data [26], the human hand mass is about 0.36 kg, plus the transradial prosthetics mass about 0.69 kg, the total mass of the below elbow hand is about 1.05 kg, which is lighter compared to the actual mass of human hand and compared to the body powered prosthesis and myoelectric prosthesis mass. The design is more comfortable since it mimics the actual mass and length of human hand.

The measurement of the average length and mass of transradial prosthetics was then designed using computer aided drafting (CAD). The dimensions followed the criteria of the subject by taking the amputation level as the main requirement. The CAD dimension gave further view on the space and the dimensions of the actuator and the sensor. The transradial prosthetics used the value of length that was based on the subject’s dimensions that were measured earlier. The design considered the level of transradial amputation and the segment of the actuator such as circuit board and motor.

### Angle for sensor detection

**Figure 4:**
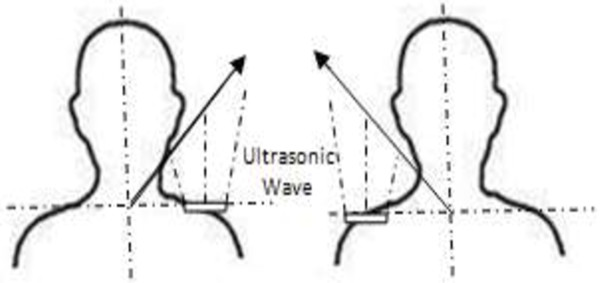
Ultrasonic wave detection between the shoulder and the head of the user.

The ultrasonic sensor wave has been programmed to detect any motion within the range of 0-15 cm and is placed on the shoulder. The motion of head to the left and right sides or the upside and down sides of the shoulder will give the detection to the ultrasonic sensor. Figure 4 shows how the motion of the head and the shoulder gives the detection to the wave of ultrasonic sensor. Generally, the ultrasonic sensor detects about 200-300 cm range if the user is inside a building or a room. The range will be reduced immediately after the head blocks the wave signal and will be continued until the range is about 15 cm [23,24]. The reason to choose the ultrasonic sensor and not other sensors such as IR sensor is because the ultrasonic sensor is more accurate in reading the receiver value. For example, it is reading in an integer value and directly can be understood by HMI (human machine interface) rather than in binary if IR sensoris used. The ultrasonic sensor also does not reflect because of the types of material or the colour of the obstacle. It will only deflect because of the other types of wave or frequency. For example, it will work very low if it is put too close between each others.

### Motion analysis for wrist movements

The experiment setup uses the combination of six MX-F20 infrared cameras into the Vicon Nexus 6.1.109 that makes up the capture system used in this study. The six MX-F20 cameras were positioned at each corner of the room and the midpoint of the room’s width. Afterwards, only object within this area may be detected by the motion signal. The calibration for using the MX-F20 was carried out by using the Vicon Nexus to give a full measurement and dimensions of the room. Each and every time the subject moves, it will give an orientation to the cameras to capture the motion. The Vicon software allows the motion to reconstruct a 3D image in space based on the calibration done. The calibration of the system needs to be calibrated each and every time during the trials. Even though the cameras are well-mounted on the wall but the area of the room may be interrupted from time to time and the detection of the cameras may be changed. Static and dynamic calibrations were carried out before the trials begin. For the static calibration, the object needs to stand at the centre of the system while for the dynamic calibration; the object needs to move from one place to another. This procedure is usually to make sure that all of the cameras work properly and the data transferred are correct [23,24].

**Figure 5:**
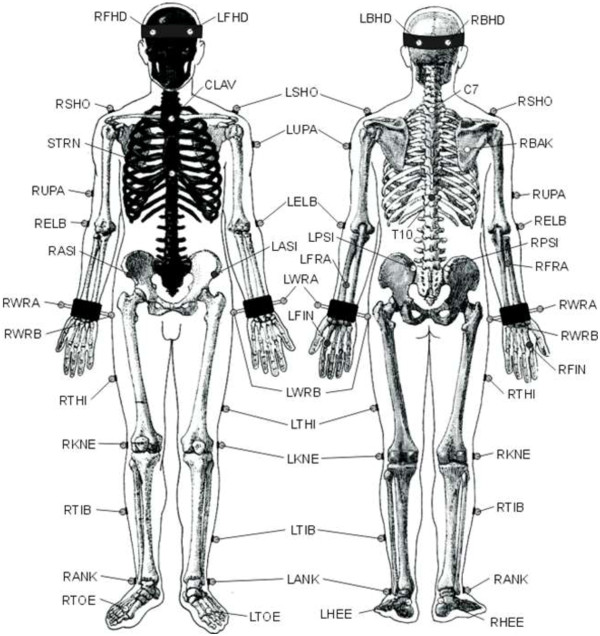
Body marker placement.

The detection of any motion within the area of the cameras depends on the reflection of the markers. The dimensions are about 14 mm in diameter and there are 32 markers positioned all around the body (refer Figure 5 and Table 3). During the experiment, the markers are placed on both of the normal and amputation subject. In order to have a full view of movement, the markers are put all around the body instead of on transradial part only. To have accurate results, the subject was advised to wear tight dress to prevent artefacts from the movement of loose dress. This is because the camera captures any movement of the markers [23,24].

**Table 3:** Marker labels, definitions and positions

LFHD	Left front head	Located approximately over the left temple
RFHD	Right front head	Located approximately over the right temple
LBHD	Left back head	Placed on the back of the head
RBHD	Right back head	Placed on the back of the head
FORE	Forehead	Middle anterior aspect of forehead
LEAR	Left ear	Left ear canal
REAR	Right ear	Right ear canal
C7	7th Cervical vertebrae	Spinous process of the 7th cervical vertebrae
T10	10th thoracic vertebrae	Spinous Process of the 10th thoracic vertebrae
CLAV	Clavicle	Jugular notch where the clavicles meet the sternum
STRN	Sternum	Xiphoid process of the sternum
RBAK	Right back	Place in the middle of the right scapula
LSHO	Left shoulder	Placed on the acromioclavicular joint
LUPA Left upper arm marker	Place on the upper arm between elbow and shoulder	
LELB	Left elbow	Place on lateral epicondyle approximating elbow joint
LMEL	Left medial elbow	Place on medial epicondyle approximating elbow
LFRA	Left forearm	Place on the lower arm between the wrist and elbow
LWRA	Left wrist marker A	Left wrist bar thumb side
LWRB	Left wrist marker B	Left wrist bar pinkie side
LFIN	Left fingers	Actually placed on the dorsum of the hand
LASI	Left ASIS	Place directly over the left anterior superior iliac spine
RASI	Right ASIS	Place directly over right anterior superior iliac spine
LPSI	Left PSIS	Place directly over left posterior superior iliac spine
RPSI	Right PSIS	Place directly over right posterior superior iliac spine
SACR	Sacral wand	Place on the skin mid-way (PSIS).
LILC	Left iliac crest	Place on the mid-superior aspect of the left iliac crest
RILC	Right iliac crest	Place on the mid-superior aspect of right iliac crest
LKNE	Left knee	Place on the lateral epicondyle of the left knee
LMKN	Left medial knee	Place on the medial epicondyle of the left knee
LTHI	Left thigh	Place over the lower lateral 1/3 surface of the thigh.
LHIP	Left hip	Superior aspect of greater trochanter
LANK	Left ankle	Place on the lateral
LMAN	Left medial ankle	Place on the medial malleolus
LTIB	Left tibial wand marker	Similar to the thigh markers
LTOE	Left toe	Place over the second metatarsal head
LHEE	Left heel	Place on the calcaneus
LHAL	Left hallux	Anterior surface of left hallux (big toe)
LMT1	Left metatarsal 1	Medial aspect of head of left metatarsal one
LMT5	Left metatarsal 5	Lateral aspect of head of left metatarsal five

The motion analysis comparison of degree rotation is the main interest in this study. The subject completed four simulated wrist general movements, which are pronation/supination and flexion/extension. Table 4 show the average of degree of movements for each motion. For the extension and flexion, the subject was asked to use the biomechatronics wrist prosthesis and moved it from the initial position to the final position. The maximum and minimum results were based on the degree of rotation.

**Table 4:** The maximum, minimum and range of motions (in degrees) during each task

	**Range of motion (ROM): biological hand**							**Range of motion (ROM): biomechatronics wrist prosthesis**						
**Type of motion**	**Test 1**	**Test 2**	**Test 3**	**Test 4**	**Test 5**	**SD**	**Average (ROM)**	**Test 1**	**Test 2**	**Test 3**	**Test 4**	**Test 5**	**SD**	**Average (ROM)**
**Flexion**	21	21.5	20.3	21.9	20.2	0.74	20.6 ± 0.74	18.7	22	24	17	19.2	2.27	22.4 ± 2.27
**Extension**	55	57	57	56	57	0.89	57.3 ± 0.89	40	39	41	41	40	0.84	41 ± 0.84
**Pronation**	55	54.6	55.2	55.7	55.4	0.41	55.7 ± 0.41	50	49.8	50.1	50	50	0.11	50.4 ± 0.11
**Supination**	50.2	47.2	48.4	51.2	50.2	1.61	50 ± 1.61	89.7	88.7	88.9	89.1	89.8	0.49	89.3 ± 0.49

The subject repeated the task five times and they were compared with the biological hand movement. Basically, the subject was asked to do each task separately, such as moving the flexion from initial position until the maximum position, moving the extension from initial position to final position, moving the supination from the initial position to final position, and moving the pronation from the initial position to final position.

## Results and discussions

Pronation of wrist motion means that the wrist part rotates about 90 degrees into body segment [12]. Normal human hand usually rotates the pronation between 85 to 90 degrees depending on the task [13]. Supination is where the forearm rotates where the palm faces up. The degree of rotation is also usually between 85 to 90 degrees depending on the task [13]. The tasks include opening a door, holding a cup, and driving a car that will bring an effect to the transradial prosthetics user [2]. Wrist flexion and extension usually occur when we open a door or we put our arm up and down. The usual range of degree for flexion is about 80° - 90° maximum and can be extended between 70° - 90°. This degree is the maximum criteria of the wrist flexion and extension task. If we want to have higher degrees then other aspects need to be controlled such as finger and elbow extension and flexion [2,13].

Table 4 simplifies the results by taking the relevant values and the averages are stated in maximum, minimum and range of each motion for the wrist movement. A problem occurred to the marker that was placed at the wrist because the position was only covered by one marker and the degree of rotation may change reliability. While doing the flexion movement, the range of normal hand motion was 20.7° in average while the prosthetics showed about 22.9° in average. The maximum of the hand flexion is usually 85°-90° but the value depends on how we stretch our muscle to reach that position [13]. The extension motion for both normal hand and prosthetic hand gave about 57° and 41°, respectively. The biomechatronics wrist prosthesis gave a lesser value due to the capability of the servo motor after several tests but the degree was already enough to do daily tasks that involve the extension motion. These two extension and flexion motions showed that the needed requirements to do daily tasks such as opening a door and filling a cup can be done.

For the pronation movement, the range of rotation of the prosthetic hand was about 55.7°. That was almost the same with the normal hand that showed 50.4° range of rotation. The pronation movement for the daily tasks is usually between the 85° - 90°. Even though the required range is higher, the degree of rotation between the normal hands with the biomechatronics wrist prosthesis was quite similar to each other. The supination movement usually only takes about 50° - 55°, which is similar to the motion for normal hand that gave about 50° of rotation [2]. But, the biomechatronics wrist prosthesis showed higher degree of rotation that almost achieved the maximum level of 89.3° of rotation.

Table 3 shows the system beginning from the input until the output of the motion analysis. The first column is about the input system that used the head motion in order to assign the input wave to the ultrasonic sensor. The degree of the head needed to bend to give the full input wave was about 45° - 55° of rotation or about 14 cm – 16 cm. The second column is about the degree of rotation of the servo motor to generate the motion. The last column is about the rotation of motion (ROM) by using motion analysis.

The biomechatronics wrist prosthesis on the other hand, gave higher degree of rotation to do the daily tasks but did not achieve the maximum degree of rotation. There were some data that showed lower degree of rotation. This was due to the lack of power supply after doing several trials. It was also due to the servo motor rotation that had its own inertia to generate a motion. The degree of rotation was then changed due to the programming system of the microcontroller and also the capability of the motor. But the objective to produce a prosthesis hand that gives similar capability of wrist movements like the normal hand was achieved.

**Figure 6:**
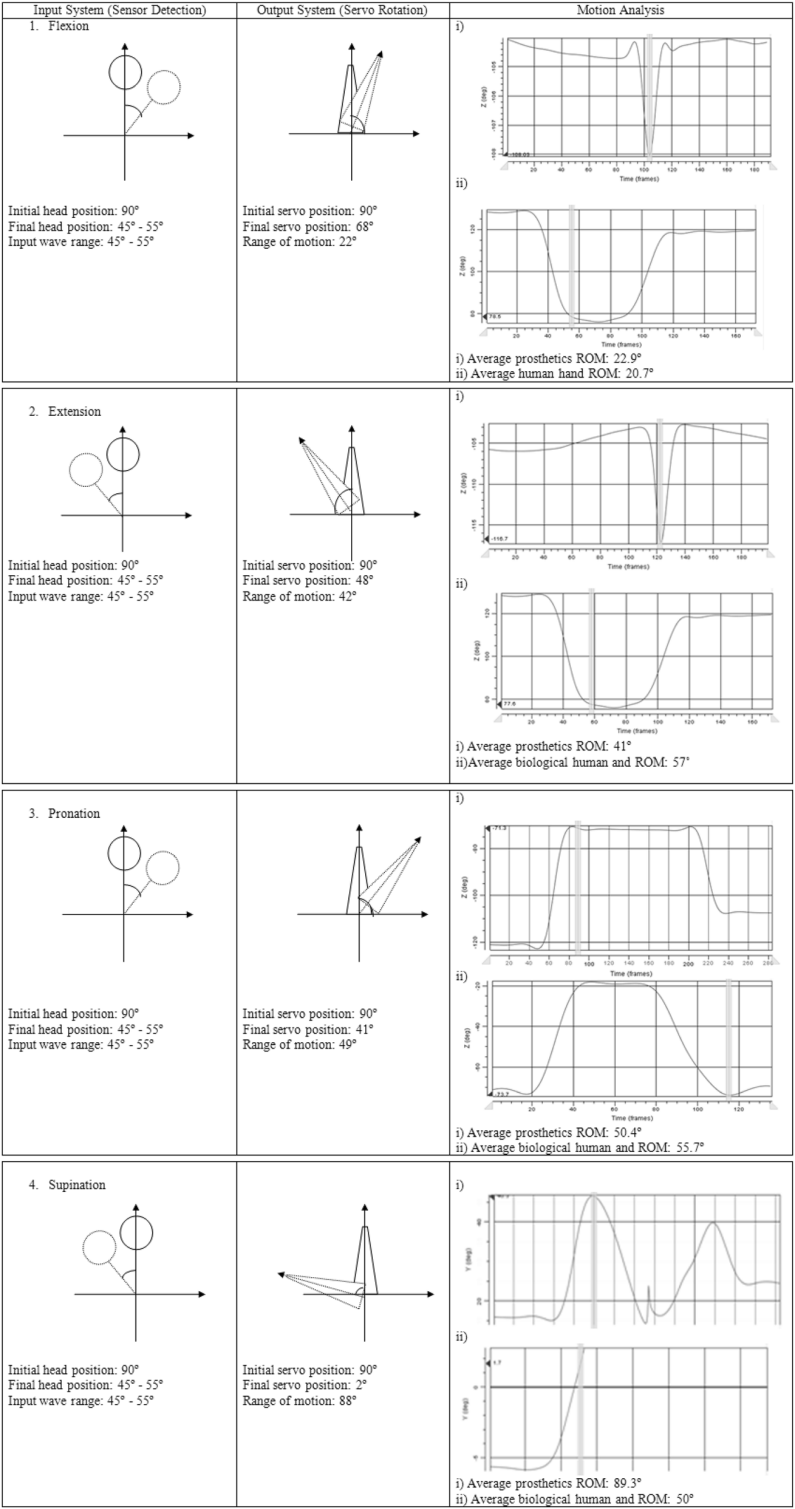
Input, output and motion analysis of biomechatronics wrist prosthesis system.

Based on Figure 6, almost all of the results gave a similar sinusoidal graph, but in terms of robotics principle, the degree of each rotation can be maximised up to 90° [28]. The 2R (two rotational) robotics theoretically can achieve up to 90°. But the results only gave the required rotation. Based on the graph in Figure 6, most of the motions done by the robotic servo motor displayed a sharp slope than the motion done by human hand.

This was due to the characteristics and speed mechanism of the motor. Human hand gave smooth and dynamic motion but the biomrchatronics wrist prosthesis gave a direct synchronous motion. That is why the slope of each motion was slightly different but at the end the results showed similar position. This is not a problem to the system since the speed and also the characteristics are greater than the human hand motion.

### Study limitations

The used of ultrasonic sensor attached at the shoulder of the subjects may interrupt the motion of head and shoulder in general condition. Any movements by the head or the shoulder may lead to generate any motion even it is not desired by the subjects. For further development, the switch may be include within the prosthetics that can be on and off whenever need as to avoid any in coincident of movements generate by the prosthesis. From the anatomical aspect, the motion of head and neck may interrupt the cervical joints and muscle between them. But comparing the effect of muscle tension given by head and neck was not as great as muscle tension for shoulder motion when using cable type prosthetics hand. Even the system cannot be consider as full achievement for developing prosthetics hand, but the placement of tension cable from body powered prosthesis to ultrasonic sensor may lead to a new finding in developing this area. At the same time the system helps reduce the power from shoulder to generate motion.

By using the servo motor to generate the pronation/supination and flexion/extension was a challenge in this research. The degree of freedom for the servo motor even though can be precisely and accurately but still giving a less rotation to achieve the maximum desire rotation as normal human hand. However, this can be counter by selecting other type of servo that gives higher rotation. But the development to replace the previous body powered prosthetics with the instalment of new technique using ultrasonic sensor and servo motor will be interesting for used list in medicine, robotics technology, rehabilitations, prosthetics and orthotics.

## Conclusion

This paper presented the design and development of new technique in generating wrist movements by using ultrasonic and servo as main sensor and actuator. The design prototype involves the anthropometrics to find the suitable measurement and size for prostheses in Malaysia. The size and measurement depends on the weight and size of each sensor, actuator and microcontroller that include inside the prosthetics hand. The replacement of ultrasonic sensor for the cable type prosthesis bring more comfort to the user and at the same time neglect the shoulder-muscle pain when generating the prosthesis.

## Abbreviations

CAD: Computer aided drafting; cm: centimeter; DC: Direct current; NM: Newton meter; V: Volt; H: Height; kg: kilogram; ROM: Rotation of motion.

## Competing interests

The authors declare that they have no competing interests.

## Authors’ contributions

NAAR and SA designed the system and the protocol, fabricated the prostheses, conducted the experiments, collected and analysed the data, discussed the results and drafted the manuscript. NAAO supervised the overall project, and helped in revising the manuscript. HG collected and analysed the data, discussed the results, wrote a part of the manuscript and helped in prosthetic fabrication. All the authors read reviewed the manuscript.

## References

[B1] Schabowsky CN Trans-radial upper extremity amputees are capable of adapting to a novel dynamic environment Exp Brain Res 2008 188 4 589 601 10.1007/s00221-008-1394-9 18443766

[B2] Carey SL Highsmith MJ Maitland ME Dubey RV Compensatory movements of transradial prosthesis users during common tasks Clin Biomech 2008 23 9 1128 1135 10.1016/j.clinbiomech.2008.05.008 18675497

[B3] Fite KB Withrow TJ Wait KW Goldfarb M Liquid-Fueled Actuation for An Anthropomorphic Upper Extremity Prosthesis Engineering in Medicine and Biology SocietyEMBS’06. 28th Annual International Conference of the IEEE 2006 5638 5642 10.1109/IEMBS.2006.259638 17947158

[B4] Fite KB Withrow TJ Wait KW Goldfarb M A Gas-Actuated Anthropomorphic Transhumeral Prosthesis Robotics and Automation, IEEE International Conference 2007 3748 3754

[B5] Controzzi M Cipriani C Carrozza MC Mechatronic Design of A Transradial Cybernetic Hand Intelligent Robots and Systems, IROS 2008 576 581

[B6] Cipriani C Controzzi M Carrozza MC Progress Towards The Development of The Smarthand Transradial Prosthesis Rehabilitation Robotics; ICORR 2009 682 687 10.1186/1743-0003-8-29 PMC3120755 21600048

[B7] Politti JC Puglisi LJ Farfán FD Prototype of a mechanical assistance device for the wrists’ flexion-extension movement J Phys Conf Ser 2007 90 1 012008

[B8] Mendoza-Vázquez JR Tlelo-Cuautle E Vázquez-Gonzalez JL Escudero-Uribe AZ Simulation of a parallel mechanical elbow with 3 DOF J App Res Tech 2009 7 2 113 123

[B9] Lee YK Shimoyama A skeletal framework artificial hand actuated by pneumatic artificial muscles In Robotics and Automation, Proceedings 1999 2 926 931

[B10] Meier R Atkins D Functional Restoration of Adults and Children with Upper Extremity Amputation Res Trends for the Twenty-First Century 30 353 360

[B11] Casolo F Cinquemani S Cocetta M Evolution of Elbow Prosthesis Transmission. Mechatronics and Its Applications ISMA 5th International Symposium 2008 1 6

[B12] Biddiss EA Chau TT Upper limb prosthesis use and abandonment: a survey of the last 25 years Prosthet orthot int 2007 31 3 236 257 10.1080/03093640600994581 17979010

[B13] Zollo L Roccella S Guglielmelli E Carrozza MC Dario P Biomechatronic design and control of an anthropomorphic artificial hand for prosthetic and robotic applications Mechatronics, IEEE/ASME Transactions 2007 12 4 418 429

[B14] Taylor CL Schwarz RJ The anatomy and mechanics of the human hand Artificial limbs 1955 2 2 22 35 13249858

[B15] Carey SL Kinematic comparison of myoelectric and body powered prostheses while performing common activities Prosthet Orthot Int 2009 33 2 179 186 10.1080/03093640802613229 19367522

[B16] Nishikawa D Yu W Yokoi H Kakazu Y EMG prosthetic hand controller discriminating ten motions using real-time learning method In Intelligent Robots and Systems IROS 1999 3 1592 1597

[B17] Lebedev MA Nicolelis M A: Brain–machine interfaces: past, present and future Trends Neurosci 2006 29 9 536 546 10.1016/j.tins.2006.07.004 16859758

[B18] Bar-Cohen Y Breazeal C Biologically inspired intelligent robots. In Smart Structures and Materials Int Soc Opt Photonics 2003 14 20

[B19] Connelly L Stoykov ME Jia Y Toro ML Kenyon RV Kamper DG Use Of A Pneumatic Glove For Hand Rehabilitation Following Stroke Engineering in Medicine and Biology Society, Annual International Conference of the IEEE 2009 2434 2437 10.1109/IEMBS.2009.5335400 19965204

[B20] Leow RS Moghavvemi M Ibrahim F An efficient low-cost real-time brain computer interface system based on SSVEP IEICE Electron Express 2010 7 5 326 331 10.1587/elex.7.326

[B21] Li G Schultz AE Kuiken TA Quantifying pattern recognition-based myoelectric control of multifunctional transradial prostheses IEEE Trans Neural Syst Rehabil Eng 2010 18 2 185 92 20071269 10.1109/TNSRE.2009.2039619 PMC3024915

[B22] Yahud S Abu Osman NA Prosthetic hand for the brain-computer interface system IFMBE 2007 15 643 646 10.1007/978-3-540-68017-8_162

[B23] Abd Razak NA Abu Osman NA Wan Abas WAB Kinematic comparison of the wrist movements that are possible with a biomechatronics wrist prosthesis and a body-powered prosthesis: a preliminary study Disabil Rehabil Assist Technol 2013 8 3 255 260 10.3109/17483107.2012.704654 22830946

[B24] Abd Razak NA Abu Osman NA Kamyab M Wan Abas WAB Gholizadeh H Satisfaction and problems experienced with wrist movements: a comparison between common body-powered prosthesis and a new biomechatronics prosthesis Am J Phys Med Rehabilin press 10.1097/PHM.0b013e3182a51fc2 24429510

[B25] Hamill J Knutzen K Biomechanical basis of human movement Aust N Z J Sociol 1995 31 3 102

[B26] Drillis R Contini R Bluestein M Body segment parameters Artificial limbs 1964 8 1 44 66 14208177

[B27] Lim TO Ding LM Zaki M Suleiman AB Fatimah S Siti S Maimunah AH Distribution of body weight, height and body mass index in a national sample of Malaysian adults Med J Malays 2000 55 1 108 128 11072496

[B28] Wang L DelPreto J Bhattacharyya S Weisz J Allen PK A Highly-Underactuated Robotic Hand With Force And Joint Angle Sensors Intelligent Robots and Systems (IROS) IEEE/RSJ International Conference on IEEE 2011 1380 1385

